# Benzene-1,3-diammonium bis­(pyridine-2,6-dicarboxyl­ato)nickelate(II) penta­hydrate

**DOI:** 10.1107/S1600536810054371

**Published:** 2011-01-15

**Authors:** Hoda Pasdar, Saghi Sadat Kashani, Hossein Aghabozorg, Behrouz Notash

**Affiliations:** a Department of Chemistry, Islamic Azad University, North Tehran Branch, Tehran, Iran; b Department of Chemistry, Shahid Beheshti University, G. C., Evin, Tehran 1983963113, Iran

## Abstract

In the title compound, (C_6_H_10_N_2_)[Ni(C_7_H_3_NO_4_)_2_]·5H_2_O, the Ni^II^ ion is six-coordinated by two N and four O atoms from two pyridine-2,6-dicarboxyl­ate ligands in a distorted octa­hedral fashion. The crystal packing is stabilized by inter­molecular O—H⋯O and N—H⋯O and weak C—H⋯O hydrogen bonds and π–π inter­actions [centroid–centroid distances = 3.4669 (19) and 3.764 (2) Å].

## Related literature

For background to proton-transfer compounds, see: Aghabozorg *et al.* (2008 ▶). For related structures, see: Aghabozorg *et al.* (2009 ▶); Beatty *et al.* (2002 ▶); Dobrzycki & Woźniak (2008 ▶); Attar Gharamaleki *et al.* (2009 ▶); Imaz *et al.* (2007 ▶); MacDon­ald *et al.* (2000 ▶, 2004 ▶); Sharif *et al.* (2007 ▶).
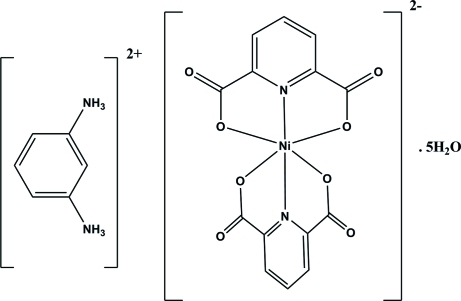


## Experimental

### 

#### Crystal data

(C_6_H_10_N_2_)[Ni(C_7_H_3_NO_4_)_2_]·5H_2_O
*M* *_r_* = 589.14
Monoclinic, 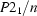

*a* = 7.5331 (15) Å
*b* = 18.085 (4) Å
*c* = 18.578 (4) Å
β = 100.90 (3)°
*V* = 2485.3 (9) Å^3^
*Z* = 4
Mo *K*α radiation
μ = 0.86 mm^−1^
*T* = 298 K
0.20 × 0.15 × 0.10 mm

#### Data collection

Stoe IPDS II diffractometer
Absorption correction: numerical (*X-RED* and *X-SHAPE*; Stoe & Cie, 2005 ▶) *T*_min_ = 0.855, *T*_max_ = 0.920
18439 measured reflections
6687 independent reflections
3608 reflections with *I* > 2σ(*I*)
*R*_int_ = 0.090

#### Refinement

*R*[*F* ^2^ > 2σ(*F* ^2^)] = 0.054
*wR*(*F* ^2^) = 0.086
*S* = 0.92
6687 reflections
407 parameters
4 restraints
H atoms treated by a mixture of independent and constrained refinement
Δρ_max_ = 0.39 e Å^−3^
Δρ_min_ = −0.36 e Å^−3^

### 

Data collection: *X-AREA* (Stoe & Cie, 2005 ▶); cell refinement: *X-AREA*; data reduction: *X-AREA*; program(s) used to solve structure: *SHELXS97* (Sheldrick, 2008 ▶); program(s) used to refine structure: *SHELXL97* (Sheldrick, 2008 ▶); molecular graphics: *ORTEP-3 for Windows* (Farrugia, 1997 ▶); software used to prepare material for publication: *WinGX* (Farrugia, 1999 ▶).

## Supplementary Material

Crystal structure: contains datablocks I, global. DOI: 10.1107/S1600536810054371/bt5430sup1.cif

Structure factors: contains datablocks I. DOI: 10.1107/S1600536810054371/bt5430Isup2.hkl

Additional supplementary materials:  crystallographic information; 3D view; checkCIF report

## Figures and Tables

**Table 1:** Hydrogen-bond geometry (Å, °)

*D*—H⋯*A*	*D*—H	H⋯*A*	*D*⋯*A*	*D*—H⋯*A*
C18—H18⋯O2^i^	0.93	2.48	3.118 (4)	126
C3—H3⋯O7^ii^	0.93	2.56	3.284 (4)	135
O13—H13*B*⋯O9^iii^	0.82 (5)	2.14 (5)	2.945 (6)	166 (5)
O13—H13*A*⋯O6^iv^	0.76 (4)	2.05 (4)	2.776 (5)	158 (6)
O12—H12*B*⋯O13^v^	0.79 (4)	2.02 (4)	2.808 (5)	176 (8)
O12—H12*A*⋯O3^vi^	0.89 (6)	1.95 (6)	2.838 (4)	174 (5)
O11—H11*B*⋯O9	0.75 (3)	2.08 (3)	2.829 (4)	173 (5)
O11—H11*A*⋯O7	0.79 (4)	2.03 (4)	2.798 (4)	163 (4)
O10—H10*B*⋯O2	0.97 (5)	1.80 (5)	2.750 (4)	168 (5)
O10—H10*A*⋯O12^vii^	0.79 (3)	2.20 (5)	2.880 (5)	146 (6)
O9—H9*B*⋯O4^ii^	0.85 (5)	2.07 (5)	2.918 (4)	172 (5)
O9—H9*A*⋯O10	0.86 (5)	1.91 (5)	2.772 (5)	176 (4)
N4—H4*C*⋯O6^viii^	0.97 (4)	1.78 (4)	2.730 (4)	167 (3)
N4—H4*B*⋯O4^vi^	0.82 (3)	2.05 (4)	2.854 (4)	168 (3)
N4—H4*A*⋯O11	0.91 (4)	1.94 (4)	2.842 (4)	173 (3)
N3—H3*C*⋯O1	0.91 (5)	1.82 (5)	2.702 (4)	164 (4)
N3—H3*B*⋯O8^vii^	0.93 (5)	1.85 (5)	2.773 (4)	173 (4)
N3—H3*A*⋯O12^vii^	0.91 (4)	2.07 (4)	2.899 (5)	152 (4)

Symmetry codes: (i) 
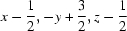
; (ii) 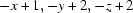; (iii) 
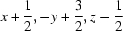
; (iv) 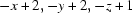; (v) 
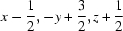
; (vi) 
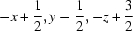
; (vii) 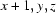; (viii) 
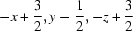
.

**Fig. 1.**
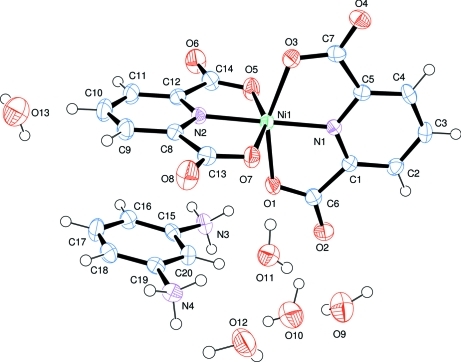
The molecular structure of the title compound with displacement ellipsoids drawn at 30% probability level.

**Fig. 2.**
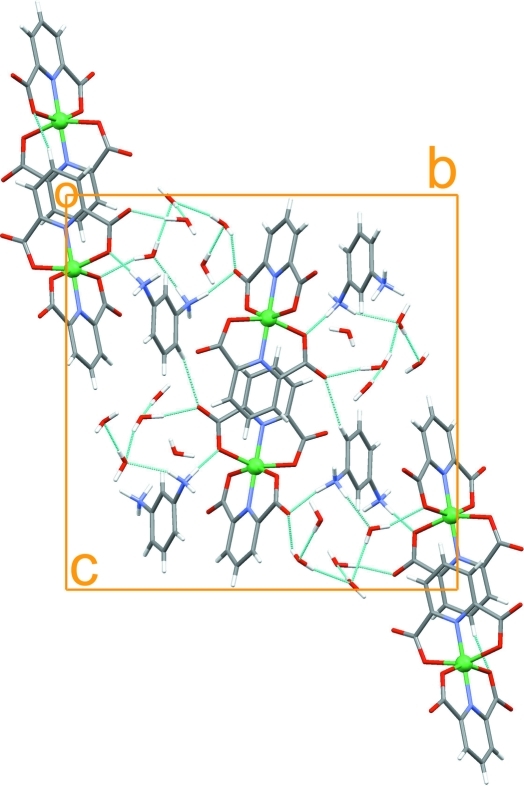
Packing diagram of the title compound viewed down the a-axis. The intermolecular O—H···O, N—H···O and C—H···O hydrogen bonds are shown as blue dashed lines.

**Fig. 3.**
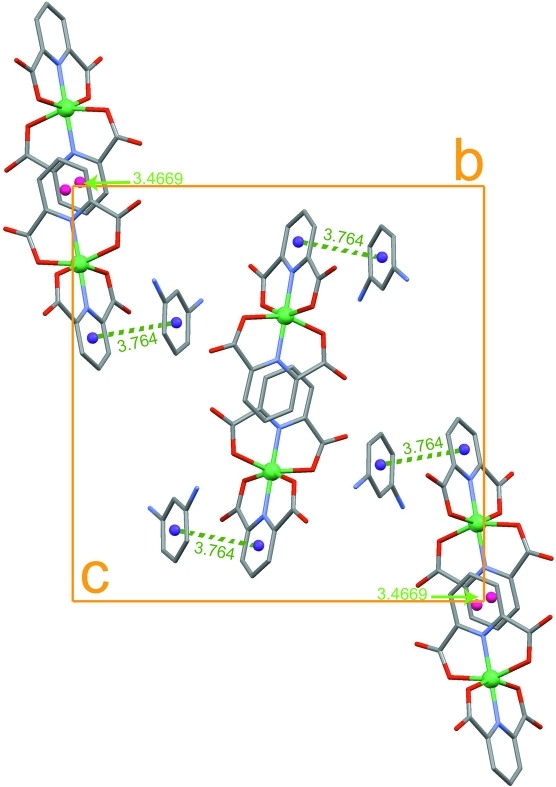
Packing diagram of the title compound viewed down the a-axis showing intermolecular π-π interactions (dashed lines). Hydrogen atoms and water molecules have been omitted for clarity.

## supplementary crystallographic information

### Comment

Pyridine-2,6-dicarboxylic acid (pydcH_2_) was commonly used as an acid in proton transfer systems (Aghabozorg *et al.* 2008). It has been reported that several proton transfer systems containing the anionic [Ni(pydc)_2_]^2-^ moiety and different cationic parts (Aghabozorg *et al.* 2009; Attar Gharamaleki *et al.* 2009; MacDonald *et al.* 2000; MacDonald *et al.* 2004; Sharif *et al.* 2007). In addition, the formation of mono (Beatty *et al.* 2002) and diprotonated benzene-1,3-diamine (Dobrzycki & Woźniak 2008; Imaz *et al.* 2007) have been observed previously.

In the title compound, (bdaH_2_)[Ni(pydc)_2_].5H_2_O, the anionic part is comprised of a Ni^II^ ion which is six-coordinated by two pyridyl nitrogen and four oxygen atoms from pydc ligands. The Ni(II) ion has a distorted octahedral geometry. (Fig. 1). Bond lengths for Ni—O and Ni—N and angles (Table 1) are in normal ranges (Aghabozorg *et al.* 2009; Attar Gharamaleki *et al.* 2009; Sharif *et al.* 2007). Crystal packing is stabilized by intermolecular O—H···O, N—H···O and weak C—H···O intermolecular hydrogen bonds which formed between [Ni(pydc)_2_]^2-^, (bdaH_2_)^2+^ and water molecules (Fig. 2 & Table 2). There are also π-π interactions between pyridine rings of pydc and between benzene ring of (bdaH_2_)^2+^ and pyridine ring of pydc molecule by distances *Cg*5···*Cg*5^ix^ and *Cg*6···*Cg*7 of 3.4669 (19) and 3.764 (2) Å, respectively. [*Cg*5, *Cg*6 and *Cg*7 are centroids of N1/C1—C6, N2/C8—C12 and C15—C20 rings, respectively. Symmetry code: (ix) 1 - *x*,2 - *y*,2 - *z*]. Intermolecular π-π interactions are shown in Fig. 3.

### Experimental

A solution of pyridine-2,6-dicarboxylic acid (pydcH_2_) (162 mg, 0.9 mmol) in 15 ml water was added to a solution of benzene-1,3-diamine (bda) (108 mg, 0.6 mmol) in 12 ml water and stirred for half an hour. Then a solution of NiCl_2_.6H_2_O (7 mg, 0.7 mmol) in 5 ml water was added to the solution of pydcH_2_ and bda. The resulted solution was stirred for 2 hrs and green crystals of the title compound were obtained after one week which were suitable for X-ray analysis (m.p 200°C).

### Refinement

The hydrogen atoms of the water molecules and  of the diammonium groups were found in a difference Fourier map and refined isotropically. The O—H bonds to H10A, H11B, H12B and H13A were refined with a distance restraint of 0.82 (4) Å. The C—H protons  were positioned geometrically and refined as riding atoms  with C–H = 0.93Å and *U*_iso_(H) = 1.2*U*_eq_(C).

### Crystal data

**Table:**

(C_6_H_10_N_2_)[Ni(C_7_H_3_NO_4_)_2_]·5H_2_O	*F*(000) = 1224.0
*M**_r_* = 589.14	*D*_x_ = 1.574 Mg m^−^^3^
Monoclinic, *P*2_1_/*n*	Mo *K*α radiation, λ = 0.71073 Å
Hall symbol: -P 2yn	Cell parameters from 6687 reflections
*a* = 7.5331 (15) Å	θ = 2.2–29.2°
*b* = 18.085 (4) Å	µ = 0.86 mm^−^^1^
*c* = 18.578 (4) Å	*T* = 298 K
β = 100.90 (3)°	Needle, green
*V* = 2485.3 (9) Å^3^	0.2 × 0.15 × 0.1 mm
*Z* = 4	

### Data collection

**Table:**

Stoe IPDS II diffractometer	6687 independent reflections
Radiation source: fine-focus sealed tube	3608 reflections with *I* > 2σ(*I*)
graphite	*R*_int_ = 0.090
Detector resolution: 0.15 mm pixels mm^-1^	θ_max_ = 29.2°, θ_min_ = 2.2°
rotation method scans	*h* = −10→9
Absorption correction: numerical [shape of crystal determined optically (*X-RED* and *X-SHAPE* (Stoe & Cie, 2005)]	*k* = −24→24
*T*_min_ = 0.855, *T*_max_ = 0.920	*l* = −25→25
18439 measured reflections	

### Refinement

**Table:**

Refinement on *F*^2^	Primary atom site location: structure-invariant direct methods
Least-squares matrix: full	Secondary atom site location: difference Fourier map
*R*[*F*^2^ > 2σ(*F*^2^)] = 0.054	Hydrogen site location: inferred from neighbouring sites
*wR*(*F*^2^) = 0.086	H atoms treated by a mixture of independent and constrained refinement
*S* = 0.92	*w* = 1/[σ^2^(*F*_o_^2^) + (0.0238*P*)^2^] where *P* = (*F*_o_^2^ + 2*F*_c_^2^)/3
6687 reflections	(Δ/σ)_max_ = 0.001
407 parameters	Δρ_max_ = 0.39 e Å^−^^3^
4 restraints	Δρ_min_ = −0.36 e Å^−^^3^

### Special details

**Table:**

Geometry. All e.s.d.'s (except the e.s.d. in the dihedral angle between two l.s. planes) are estimated using the full covariance matrix. The cell e.s.d.'s are taken into account individually in the estimation of e.s.d.'s in distances, angles and torsion angles; correlations between e.s.d.'s in cell parameters are only used when they are defined by crystal symmetry. An approximate (isotropic) treatment of cell e.s.d.'s is used for estimating e.s.d.'s involving l.s. planes.
Refinement. Refinement of *F*^2^ against ALL reflections. The weighted *R*-factor *wR* and goodness of fit *S* are based on *F*^2^, conventional *R*-factors *R* are based on *F*, with *F* set to zero for negative *F*^2^. The threshold expression of *F*^2^ > σ(*F*^2^) is used only for calculating *R*-factors(gt) *etc*. and is not relevant to the choice of reflections for refinement. *R*-factors based on *F*^2^ are statistically about twice as large as those based on *F*, and *R*- factors based on ALL data will be even larger.

### Fractional atomic coordinates and isotropic or equivalent isotropic displacement parameters (Å^2^)

**Table:**

	*x*	*y*	*z*	*U*_iso_*/*U*_eq_	
O12	0.0352 (4)	0.65115 (16)	0.82305 (17)	0.0522 (8)	
Ni1	0.64385 (6)	0.98344 (2)	0.81233 (2)	0.02316 (10)	
N1	0.6858 (3)	1.00256 (12)	0.91843 (12)	0.0203 (5)	
N2	0.6146 (3)	0.96578 (13)	0.70636 (12)	0.0225 (6)	
O7	0.4041 (3)	0.91734 (12)	0.79023 (11)	0.0312 (5)	
O3	0.4988 (3)	1.08346 (11)	0.81671 (11)	0.0305 (5)	
O5	0.8722 (3)	1.03845 (12)	0.78742 (11)	0.0368 (6)	
C11	0.7284 (5)	0.97699 (19)	0.59762 (16)	0.0354 (8)	
H11	0.8166	0.9944	0.5730	0.042*	
C12	0.7401 (4)	0.99113 (17)	0.67087 (15)	0.0262 (7)	
O1	0.8002 (3)	0.89096 (11)	0.85564 (11)	0.0315 (5)	
C1	0.7845 (4)	0.95527 (15)	0.96399 (15)	0.0200 (6)	
C5	0.6176 (4)	1.06446 (15)	0.94246 (15)	0.0227 (7)	
N3	0.9677 (4)	0.80203 (19)	0.77250 (17)	0.0290 (6)	
C3	0.7580 (4)	1.03320 (17)	1.06483 (16)	0.0277 (7)	
H3	0.7827	1.0437	1.1147	0.033*	
C4	0.6531 (4)	1.08166 (17)	1.01596 (16)	0.0275 (7)	
H4	0.6078	1.1249	1.0328	0.033*	
C2	0.8252 (4)	0.96944 (16)	1.03883 (15)	0.0272 (7)	
H2	0.8960	0.9368	1.0707	0.033*	
C8	0.4727 (4)	0.92723 (16)	0.67186 (16)	0.0255 (7)	
C15	0.8119 (4)	0.77521 (16)	0.71966 (16)	0.0241 (7)	
C7	0.5013 (4)	1.10803 (16)	0.88110 (17)	0.0256 (7)	
C20	0.6666 (4)	0.74641 (17)	0.74619 (17)	0.0261 (7)	
H20	0.6665	0.7454	0.7962	0.031*	
C16	0.8142 (4)	0.77784 (17)	0.64577 (17)	0.0289 (7)	
H16	0.9119	0.7982	0.6287	0.035*	
O4	0.4185 (3)	1.16332 (11)	0.89702 (12)	0.0322 (5)	
O2	0.9292 (4)	0.83930 (13)	0.96180 (12)	0.0431 (6)	
O8	0.2157 (3)	0.86339 (14)	0.69685 (13)	0.0467 (7)	
O6	1.0038 (3)	1.06724 (13)	0.69308 (13)	0.0468 (7)	
C19	0.5220 (4)	0.71920 (16)	0.69634 (16)	0.0238 (7)	
N4	0.3701 (4)	0.68770 (17)	0.72430 (17)	0.0275 (6)	
C18	0.5215 (4)	0.72028 (17)	0.62201 (17)	0.0303 (8)	
H18	0.4235	0.7015	0.5890	0.036*	
C6	0.8438 (4)	0.88880 (17)	0.92559 (16)	0.0266 (7)	
C14	0.8847 (4)	1.03584 (17)	0.72077 (17)	0.0311 (8)	
C17	0.6668 (5)	0.74933 (19)	0.59727 (17)	0.0369 (8)	
H17	0.6669	0.7500	0.5472	0.044*	
C9	0.4516 (5)	0.91218 (18)	0.59783 (17)	0.0334 (8)	
H9	0.3513	0.8863	0.5734	0.040*	
C10	0.5841 (5)	0.93673 (19)	0.56106 (18)	0.0394 (9)	
H10	0.5756	0.9260	0.5116	0.047*	
C13	0.3517 (4)	0.90100 (17)	0.72284 (16)	0.0271 (7)	
O11	0.3389 (4)	0.77842 (17)	0.84618 (15)	0.0402 (7)	
O9	0.5261 (5)	0.72860 (19)	0.98420 (18)	0.0583 (9)	
O10	0.8429 (5)	0.6926 (2)	0.93695 (19)	0.0649 (9)	
O13	0.8277 (5)	0.9051 (2)	0.4247 (2)	0.0646 (9)	
H11A	0.360 (6)	0.820 (2)	0.839 (2)	0.057 (15)*	
H11B	0.381 (6)	0.765 (2)	0.8838 (19)	0.062 (15)*	
H12B	0.114 (8)	0.633 (4)	0.852 (3)	0.15 (3)*	
H13A	0.879 (7)	0.923 (3)	0.398 (3)	0.11 (3)*	
H12A	0.025 (7)	0.627 (3)	0.781 (3)	0.103 (19)*	
H9B	0.552 (7)	0.761 (3)	1.018 (3)	0.09 (2)*	
H9A	0.628 (7)	0.718 (2)	0.972 (2)	0.067 (17)*	
H10A	0.857 (8)	0.670 (3)	0.902 (2)	0.10 (2)*	
H13B	0.894 (7)	0.873 (3)	0.446 (3)	0.08 (2)*	
H10B	0.882 (7)	0.744 (3)	0.940 (3)	0.095 (18)*	
H3A	1.023 (6)	0.763 (2)	0.797 (2)	0.063 (14)*	
H3B	1.051 (6)	0.826 (2)	0.750 (2)	0.056 (13)*	
H3C	0.932 (6)	0.834 (2)	0.805 (2)	0.061 (13)*	
H4C	0.398 (5)	0.641 (2)	0.750 (2)	0.054 (12)*	
H4A	0.351 (5)	0.716 (2)	0.763 (2)	0.045 (11)*	
H4B	0.279 (5)	0.6851 (18)	0.6925 (19)	0.034 (10)*	

### Atomic displacement parameters (Å^2^)

**Table:**

	*U*^11^	*U*^22^	*U*^33^	*U*^12^	*U*^13^	*U*^23^
O12	0.064 (2)	0.0460 (17)	0.0440 (17)	0.0030 (15)	0.0021 (16)	−0.0136 (14)
Ni1	0.0261 (2)	0.02501 (18)	0.01821 (16)	−0.0019 (2)	0.00388 (14)	−0.00170 (18)
N1	0.0188 (12)	0.0206 (14)	0.0222 (12)	−0.0019 (9)	0.0056 (10)	−0.0015 (9)
N2	0.0244 (13)	0.0238 (14)	0.0202 (12)	−0.0022 (10)	0.0065 (10)	−0.0012 (10)
O7	0.0347 (13)	0.0370 (13)	0.0238 (11)	−0.0078 (10)	0.0100 (10)	−0.0045 (9)
O3	0.0346 (13)	0.0301 (12)	0.0247 (11)	0.0043 (10)	0.0004 (10)	0.0004 (9)
O5	0.0391 (14)	0.0434 (14)	0.0279 (11)	−0.0156 (11)	0.0059 (10)	−0.0076 (10)
C11	0.047 (2)	0.0370 (18)	0.0253 (15)	−0.0073 (18)	0.0145 (14)	−0.0003 (15)
C12	0.0315 (16)	0.0238 (16)	0.0247 (14)	−0.0044 (14)	0.0087 (12)	0.0014 (13)
O1	0.0398 (14)	0.0310 (12)	0.0236 (11)	0.0110 (10)	0.0055 (10)	−0.0036 (9)
C1	0.0137 (14)	0.0242 (15)	0.0232 (14)	−0.0027 (12)	0.0065 (12)	0.0009 (12)
C5	0.0205 (15)	0.0215 (15)	0.0259 (15)	−0.0013 (12)	0.0037 (13)	0.0000 (12)
N3	0.0201 (15)	0.0389 (18)	0.0281 (15)	−0.0029 (14)	0.0049 (13)	−0.0087 (14)
C3	0.0271 (16)	0.0371 (19)	0.0194 (14)	−0.0066 (14)	0.0055 (13)	−0.0057 (12)
C4	0.0247 (16)	0.0303 (17)	0.0298 (16)	−0.0002 (13)	0.0108 (13)	−0.0110 (13)
C2	0.0287 (16)	0.0288 (18)	0.0222 (14)	−0.0009 (13)	0.0003 (13)	0.0027 (12)
C8	0.0308 (18)	0.0221 (15)	0.0228 (15)	−0.0016 (13)	0.0033 (13)	0.0001 (12)
C15	0.0191 (15)	0.0245 (16)	0.0275 (16)	0.0021 (13)	0.0013 (13)	−0.0072 (13)
C7	0.0231 (16)	0.0222 (16)	0.0300 (17)	−0.0034 (13)	0.0012 (13)	−0.0005 (13)
C20	0.0230 (16)	0.0331 (17)	0.0223 (15)	−0.0011 (14)	0.0045 (13)	−0.0028 (13)
C16	0.0272 (17)	0.0323 (18)	0.0291 (17)	−0.0044 (14)	0.0102 (14)	−0.0026 (14)
O4	0.0273 (12)	0.0288 (12)	0.0397 (13)	0.0071 (10)	0.0046 (10)	−0.0025 (10)
O2	0.0580 (17)	0.0336 (13)	0.0354 (13)	0.0184 (12)	0.0028 (12)	0.0041 (10)
O8	0.0359 (15)	0.0651 (17)	0.0383 (14)	−0.0234 (13)	0.0050 (12)	−0.0055 (12)
O6	0.0472 (16)	0.0525 (16)	0.0451 (15)	−0.0244 (13)	0.0199 (12)	−0.0063 (12)
C19	0.0195 (15)	0.0224 (15)	0.0298 (16)	0.0021 (12)	0.0055 (13)	−0.0021 (13)
N4	0.0202 (15)	0.0293 (16)	0.0323 (16)	−0.0033 (12)	0.0029 (13)	−0.0012 (13)
C18	0.0294 (18)	0.0313 (18)	0.0282 (16)	−0.0047 (14)	0.0001 (14)	−0.0062 (13)
C6	0.0266 (17)	0.0269 (17)	0.0266 (16)	−0.0008 (14)	0.0056 (14)	−0.0010 (13)
C14	0.0299 (18)	0.0325 (19)	0.0337 (17)	−0.0066 (14)	0.0132 (14)	−0.0037 (14)
C17	0.042 (2)	0.047 (2)	0.0215 (16)	−0.0046 (17)	0.0054 (16)	−0.0021 (15)
C9	0.036 (2)	0.0371 (19)	0.0248 (16)	−0.0068 (15)	0.0012 (15)	−0.0032 (14)
C10	0.054 (2)	0.044 (2)	0.0226 (17)	−0.0075 (18)	0.0128 (16)	−0.0024 (15)
C13	0.0239 (17)	0.0287 (17)	0.0299 (17)	−0.0004 (14)	0.0082 (14)	0.0003 (13)
O11	0.0474 (17)	0.0362 (16)	0.0357 (16)	−0.0017 (13)	0.0051 (13)	0.0008 (13)
O9	0.059 (2)	0.068 (2)	0.0464 (19)	0.0053 (18)	0.0066 (17)	−0.0100 (16)
O10	0.077 (2)	0.058 (2)	0.063 (2)	0.0039 (18)	0.0247 (18)	−0.0084 (18)
O13	0.060 (2)	0.082 (3)	0.059 (2)	−0.001 (2)	0.0275 (19)	0.0045 (19)

### Geometric parameters (Å, °)

**Table:**

O12—H12B	0.79 (4)	C2—H2	0.9300
O12—H12A	0.89 (6)	C8—C9	1.381 (4)
Ni1—N2	1.965 (2)	C8—C13	1.510 (5)
Ni1—N1	1.967 (2)	C15—C16	1.377 (4)
Ni1—O5	2.113 (2)	C15—C20	1.384 (4)
Ni1—O1	2.115 (2)	C7—O4	1.244 (4)
Ni1—O3	2.123 (2)	C20—C19	1.380 (4)
Ni1—O7	2.140 (2)	C20—H20	0.9300
N1—C1	1.328 (3)	C16—C17	1.390 (4)
N1—C5	1.343 (4)	C16—H16	0.9300
N2—C12	1.332 (4)	O2—C6	1.226 (3)
N2—C8	1.334 (4)	O8—C13	1.248 (4)
O7—C13	1.274 (3)	O6—C14	1.251 (4)
O3—C7	1.273 (4)	C19—C18	1.381 (4)
O5—C14	1.260 (4)	C19—N4	1.459 (4)
C11—C12	1.371 (4)	N4—H4C	0.97 (4)
C11—C10	1.376 (5)	N4—H4A	0.91 (4)
C11—H11	0.9300	N4—H4B	0.82 (3)
C12—C14	1.522 (4)	C18—C17	1.370 (5)
O1—C6	1.279 (3)	C18—H18	0.9300
C1—C2	1.390 (4)	C17—H17	0.9300
C1—C6	1.508 (4)	C9—C10	1.385 (5)
C5—C4	1.376 (4)	C9—H9	0.9300
C5—C7	1.520 (4)	C10—H10	0.9300
N3—C15	1.463 (4)	O11—H11A	0.79 (4)
N3—H3A	0.91 (4)	O11—H11B	0.75 (3)
N3—H3B	0.93 (5)	O9—H9B	0.85 (5)
N3—H3C	0.91 (5)	O9—H9A	0.86 (5)
C3—C2	1.383 (4)	O10—H10A	0.79 (3)
C3—C4	1.395 (4)	O10—H10B	0.97 (5)
C3—H3	0.9300	O13—H13A	0.76 (4)
C4—H4	0.9300	O13—H13B	0.82 (5)
			
H12B—O12—H12A	109 (6)	C3—C2—H2	120.7
N2—Ni1—N1	177.13 (11)	C1—C2—H2	120.7
N2—Ni1—O5	78.39 (10)	N2—C8—C9	120.8 (3)
N1—Ni1—O5	98.84 (9)	N2—C8—C13	112.7 (3)
N2—Ni1—O1	101.62 (9)	C9—C8—C13	126.4 (3)
N1—Ni1—O1	77.61 (9)	C16—C15—C20	121.9 (3)
O5—Ni1—O1	92.17 (9)	C16—C15—N3	119.8 (3)
N2—Ni1—O3	102.47 (9)	C20—C15—N3	118.3 (3)
N1—Ni1—O3	78.34 (9)	O4—C7—O3	125.8 (3)
O5—Ni1—O3	93.06 (9)	O4—C7—C5	118.9 (3)
O1—Ni1—O3	155.91 (8)	O3—C7—C5	115.3 (3)
N2—Ni1—O7	77.66 (10)	C19—C20—C15	118.2 (3)
N1—Ni1—O7	105.08 (9)	C19—C20—H20	120.9
O5—Ni1—O7	156.01 (8)	C15—C20—H20	120.9
O1—Ni1—O7	91.16 (9)	C15—C16—C17	118.3 (3)
O3—Ni1—O7	93.54 (9)	C15—C16—H16	120.8
C1—N1—C5	121.9 (2)	C17—C16—H16	120.8
C1—N1—Ni1	119.34 (19)	C20—C19—C18	121.2 (3)
C5—N1—Ni1	118.74 (18)	C20—C19—N4	118.2 (3)
C12—N2—C8	121.4 (3)	C18—C19—N4	120.5 (3)
C12—N2—Ni1	118.97 (19)	C19—N4—H4C	114 (2)
C8—N2—Ni1	119.6 (2)	C19—N4—H4A	108 (2)
C13—O7—Ni1	114.3 (2)	H4C—N4—H4A	99 (3)
C7—O3—Ni1	114.67 (18)	C19—N4—H4B	112 (3)
C14—O5—Ni1	114.38 (19)	H4C—N4—H4B	112 (3)
C12—C11—C10	119.0 (3)	H4A—N4—H4B	111 (3)
C12—C11—H11	120.5	C17—C18—C19	119.3 (3)
C10—C11—H11	120.5	C17—C18—H18	120.3
N2—C12—C11	120.6 (3)	C19—C18—H18	120.3
N2—C12—C14	112.0 (2)	O2—C6—O1	125.9 (3)
C11—C12—C14	127.4 (3)	O2—C6—C1	119.6 (3)
C6—O1—Ni1	115.35 (19)	O1—C6—C1	114.5 (3)
N1—C1—C2	120.7 (3)	O6—C14—O5	125.4 (3)
N1—C1—C6	113.1 (2)	O6—C14—C12	118.5 (3)
C2—C1—C6	126.2 (3)	O5—C14—C12	116.1 (3)
N1—C5—C4	120.3 (3)	C18—C17—C16	121.1 (3)
N1—C5—C7	112.7 (2)	C18—C17—H17	119.5
C4—C5—C7	127.1 (3)	C16—C17—H17	119.5
C15—N3—H3A	108 (3)	C8—C9—C10	118.0 (3)
C15—N3—H3B	112 (2)	C8—C9—H9	121.0
H3A—N3—H3B	108 (4)	C10—C9—H9	121.0
C15—N3—H3C	111 (3)	C11—C10—C9	120.1 (3)
H3A—N3—H3C	109 (4)	C11—C10—H10	119.9
H3B—N3—H3C	109 (4)	C9—C10—H10	119.9
C2—C3—C4	119.8 (3)	O8—C13—O7	125.8 (3)
C2—C3—H3	120.1	O8—C13—C8	118.5 (3)
C4—C3—H3	120.1	O7—C13—C8	115.6 (3)
C5—C4—C3	118.9 (3)	H11A—O11—H11B	113 (4)
C5—C4—H4	120.6	H9B—O9—H9A	105 (5)
C3—C4—H4	120.6	H10A—O10—H10B	117 (6)
C3—C2—C1	118.5 (3)	H13A—O13—H13B	106 (6)
			
N2—Ni1—N1—C1	−74 (2)	Ni1—N1—C5—C4	−176.3 (2)
O5—Ni1—N1—C1	−89.1 (2)	C1—N1—C5—C7	−177.0 (3)
O1—Ni1—N1—C1	1.2 (2)	Ni1—N1—C5—C7	4.9 (3)
O3—Ni1—N1—C1	179.6 (2)	N1—C5—C4—C3	−1.0 (5)
O7—Ni1—N1—C1	89.0 (2)	C7—C5—C4—C3	177.5 (3)
N2—Ni1—N1—C5	104 (2)	C2—C3—C4—C5	0.4 (5)
O5—Ni1—N1—C5	89.0 (2)	C4—C3—C2—C1	−0.4 (5)
O1—Ni1—N1—C5	179.3 (2)	N1—C1—C2—C3	1.1 (4)
O3—Ni1—N1—C5	−2.3 (2)	C6—C1—C2—C3	−179.5 (3)
O7—Ni1—N1—C5	−92.9 (2)	C12—N2—C8—C9	0.2 (4)
N1—Ni1—N2—C12	−14 (2)	Ni1—N2—C8—C9	−178.1 (2)
O5—Ni1—N2—C12	2.1 (2)	C12—N2—C8—C13	177.5 (3)
O1—Ni1—N2—C12	−87.8 (2)	Ni1—N2—C8—C13	−0.8 (3)
O3—Ni1—N2—C12	92.7 (2)	Ni1—O3—C7—O4	−176.1 (3)
O7—Ni1—N2—C12	−176.4 (2)	Ni1—O3—C7—C5	4.0 (3)
N1—Ni1—N2—C8	164.8 (19)	N1—C5—C7—O4	174.2 (3)
O5—Ni1—N2—C8	−179.6 (2)	C4—C5—C7—O4	−4.4 (5)
O1—Ni1—N2—C8	90.5 (2)	N1—C5—C7—O3	−5.9 (4)
O3—Ni1—N2—C8	−89.0 (2)	C4—C5—C7—O3	175.5 (3)
O7—Ni1—N2—C8	1.9 (2)	C16—C15—C20—C19	0.6 (5)
N2—Ni1—O7—C13	−2.9 (2)	N3—C15—C20—C19	−178.2 (3)
N1—Ni1—O7—C13	178.0 (2)	C20—C15—C16—C17	−1.2 (5)
O5—Ni1—O7—C13	−6.6 (3)	N3—C15—C16—C17	177.6 (3)
O1—Ni1—O7—C13	−104.5 (2)	C15—C20—C19—C18	0.2 (4)
O3—Ni1—O7—C13	99.1 (2)	C15—C20—C19—N4	179.0 (3)
N2—Ni1—O3—C7	−178.4 (2)	C20—C19—C18—C17	−0.4 (5)
N1—Ni1—O3—C7	−1.2 (2)	N4—C19—C18—C17	−179.2 (3)
O5—Ni1—O3—C7	−99.6 (2)	Ni1—O1—C6—O2	177.8 (3)
O1—Ni1—O3—C7	2.7 (4)	Ni1—O1—C6—C1	−3.4 (3)
O7—Ni1—O3—C7	103.5 (2)	N1—C1—C6—O2	−176.8 (3)
N2—Ni1—O5—C14	0.6 (2)	C2—C1—C6—O2	3.7 (5)
N1—Ni1—O5—C14	179.8 (2)	N1—C1—C6—O1	4.4 (4)
O1—Ni1—O5—C14	102.0 (2)	C2—C1—C6—O1	−175.1 (3)
O3—Ni1—O5—C14	−101.6 (2)	Ni1—O5—C14—O6	176.6 (3)
O7—Ni1—O5—C14	4.2 (4)	Ni1—O5—C14—C12	−2.7 (3)
C8—N2—C12—C11	−1.2 (4)	N2—C12—C14—O6	−175.0 (3)
Ni1—N2—C12—C11	177.1 (2)	C11—C12—C14—O6	3.9 (5)
C8—N2—C12—C14	177.8 (3)	N2—C12—C14—O5	4.3 (4)
Ni1—N2—C12—C14	−3.9 (3)	C11—C12—C14—O5	−176.7 (3)
C10—C11—C12—N2	0.6 (5)	C19—C18—C17—C16	−0.2 (5)
C10—C11—C12—C14	−178.3 (3)	C15—C16—C17—C18	0.9 (5)
N2—Ni1—O1—C6	178.6 (2)	N2—C8—C9—C10	1.5 (5)
N1—Ni1—O1—C6	1.5 (2)	C13—C8—C9—C10	−175.4 (3)
O5—Ni1—O1—C6	100.0 (2)	C12—C11—C10—C9	1.1 (5)
O3—Ni1—O1—C6	−2.4 (4)	C8—C9—C10—C11	−2.1 (5)
O7—Ni1—O1—C6	−103.7 (2)	Ni1—O7—C13—O8	−179.7 (3)
C5—N1—C1—C2	−1.8 (4)	Ni1—O7—C13—C8	3.3 (3)
Ni1—N1—C1—C2	176.3 (2)	N2—C8—C13—O8	−179.0 (3)
C5—N1—C1—C6	178.8 (3)	C9—C8—C13—O8	−1.9 (5)
Ni1—N1—C1—C6	−3.2 (3)	N2—C8—C13—O7	−1.8 (4)
C1—N1—C5—C4	1.7 (4)	C9—C8—C13—O7	175.3 (3)

### Hydrogen-bond geometry (Å, °)

**Table:**

*D*—H···*A*	*D*—H	H···*A*	*D*···*A*	*D*—H···*A*
C18—H18···O2^i^	0.93	2.48	3.118 (4)	126.
C3—H3···O7^ii^	0.93	2.56	3.284 (4)	135.
O13—H13B···O9^iii^	0.82 (5)	2.14 (5)	2.945 (6)	166 (5)
O13—H13A···O6^iv^	0.76 (4)	2.05 (4)	2.776 (5)	158 (6)
O12—H12B···O13^v^	0.79 (4)	2.02 (4)	2.808 (5)	176 (8)
O12—H12A···O3^vi^	0.89 (6)	1.95 (6)	2.838 (4)	174 (5)
O11—H11B···O9	0.75 (3)	2.08 (3)	2.829 (4)	173 (5)
O11—H11A···O7	0.79 (4)	2.03 (4)	2.798 (4)	163 (4)
O10—H10B···O2	0.97 (5)	1.80 (5)	2.750 (4)	168 (5)
O10—H10A···O12^vii^	0.79 (3)	2.20 (5)	2.880 (5)	146 (6)
O9—H9B···O4^ii^	0.85 (5)	2.07 (5)	2.918 (4)	172 (5)
O9—H9A···O10	0.86 (5)	1.91 (5)	2.772 (5)	176 (4)
N4—H4C···O6^viii^	0.97 (4)	1.78 (4)	2.730 (4)	167 (3)
N4—H4B···O4^vi^	0.82 (3)	2.05 (4)	2.854 (4)	168 (3)
N4—H4A···O11	0.91 (4)	1.94 (4)	2.842 (4)	173 (3)
N3—H3C···O1	0.91 (5)	1.82 (5)	2.702 (4)	164 (4)
N3—H3B···O8^vii^	0.93 (5)	1.85 (5)	2.773 (4)	173 (4)
N3—H3A···O12^vii^	0.91 (4)	2.07 (4)	2.899 (5)	152 (4)

Symmetry codes: (i) *x*−1/2, −*y*+3/2, *z*−1/2; (ii) −*x*+1, −*y*+2, −*z*+2; (iii) *x*+1/2, −*y*+3/2, *z*−1/2; (iv) −*x*+2, −*y*+2, −*z*+1; (v) *x*−1/2, −*y*+3/2, *z*+1/2; (vi) −*x*+1/2, *y*−1/2, −*z*+3/2; (vii) *x*+1, *y*, *z*; (viii) −*x*+3/2, *y*−1/2, −*z*+3/2.
